# Enhancing Knowledge in Informal Settlements: Assessing Health Beliefs and Behaviors in Nigeria: A cross-sectional survey assessment of perceptions, practices, and resources in underserved urban communities in Lagos

**DOI:** 10.5334/aogh.2648

**Published:** 2020-09-22

**Authors:** Daniel Y. Liu, Andrew W. Maki, Anna Maitland, Elise R. Meyer, Juliet S. Sorensen, Shannon Galvin

**Affiliations:** 1 Feinberg School of Medicine, Chicago, IL, US; 2 Justice & Empowerment Initiatives Inc., Lagos, NG; 3 Northwestern University Pritzker School of Law, Chicago, IL, US

## Abstract

**Background:**

Community Health Education (CHE) programs have been shown to be effective in relieving the burden on healthcare systems in Sub-Saharan Africa.

**Objective:**

This project aimed to determine the baseline level of health literacy, behavioral practices, and accessibility to resources in a set of 16 informal settlements located around Lagos, Nigeria in order to identify topics that should be emphasized in a new teaching curriculum directed at local Community Health Educators.

**Methods:**

In June of 2017, a unique cross-sectional survey composed of 37 questions was conducted in informal settlements around Lagos. Sites selected were areas in which future CHE trainings were planned to take place and survey participants were chosen by trained community health educators based on convenience sampling with snowball effect. Survey questions included both multiple-choice and open-ended questions and were asked in the local language. We collected demographic information and assessed health literacy, health behaviors, and community practices. Results were analyzed using descriptive statistics to assess for differences between demographic groups.

**Findings:**

Our survey collected 348 total responses. Respondents displayed a high level of knowledge regarding the benefits of hand washing (97.1%) and childhood immunizations (81.0%). Knowledge around infectious diseases and reproductive health was lower, including a large proportion of people (50%) incorrectly indicating that HIV could be spread through a mosquito bite. Malaria was reported to be the most prevalent disease affecting both adults (40.0%) and children (58.3%). Health access was limited, with most people not reporting access to a nearby health center (55.8%).

**Conclusions:**

Areas of knowledge that should be emphasized in future versions of CHE training curricula include infectious diseases, reproductive health, and reinforcement of the importance of sanitation and clean water. The curriculum should address the reality of limited health access and develop strategies to improve this.

## Background

Home to an estimated 214 million inhabitants, Nigeria is the most populous nation in Africa and continues to grow at a rapid rate [1]. Despite modern advancements, over one quarter of the global burden of malaria, a disease that is both preventable and treatable, is found in Nigeria [2]. A high prevalence of diarrheal disease and typhoid is also concerning as these contribute to a high child mortality rate [3,4]. Increasing awareness about the benefits of vaccinations and basic sanitary behaviors would attenuate the burden that these diseases place on communities.

One strategy that has been shown to improve health systems in other sub-Saharan African countries is Community Health Education (CHE). These programs train community members to assist health professionals in promoting health literacy and connecting neglected populations to basic services [5,6,7]. As defined by the World Health Organization (WHO) health promotion glossary, health literacy “represents the cognitive and social skills which determine the motivation and ability of individuals to gain access to, understand and use information in ways which promote and maintain good health [8].” CHE programs can serve as transformative vessels for their regions by communicating knowledge to their populations within a cultural context that is familiar and accepted [9,10,11]. Furthermore, targeting health education efforts towards at-risk populations has been shown to have an especially pronounced impact on improving the health of women and children [12,13].

Despite becoming one of the world’s fastest expanding economies, the benefits of Nigeria’s prosperity have fallen well short of equitable distribution [14]. The impoverished conditions in which millions of Nigerians live have resulted in citizens being forced into low-income settlements that increase the risk of spreading disease, are devoid of sanitation standards, and lack accessible healthcare services [15]. These slums do not have reliable access to sanitary drinking sources and contain obstructed drains that result in pools of standing water that increase disease proliferation [16,17]. Previous studies have already demonstrated the link between higher health literacy and improved outcomes in Nigerian communities for malaria and maternal health [18,19]. Programs that emphasize education and promote the utilization of existing health infrastructure will improve health literacy, and eventually health outcomes, in Nigeria.

### Objectives

Our work was conducted through Access to Health (ATH), a partnership between graduate schools at Northwestern University and the Justice & Empowerment Initiatives (JEI). This partnership is an interdisciplinary global community health project that brings law, public health, medical, and business faculty as well as graduate students together with communities, health advocates, government, university institutions, and human rights organizations in other countries. ATH engages in global health issues by working directly with communities and local NGOs to 1) develop targeted and sustainable projects that respond to the needs of health-poor communities, and 2) teach students how to engage in interdisciplinary, transnational partnerships that encourage global citizenship and understanding.

ATH’s local partners in Nigeria are Justice and Empowerment Initiatives – Nigeria (JEI), a civil society organization working in Nigerian urban informal settlements – and the Nigerian Slum/Informal Settlement Federation (Federation), an organization composed of community-level savings groups in slums and informal settlements in Lagos, Port Harcourt, and other Nigerian cities. The Federation collects community-generated data to feed into advocacy around informal settlement communities’ collective priorities, including land tenure and access to basic services such as electricity, potable water, elementary education, and health. While supporting other Federation activities, JEI helps equip the urban poor with the knowledge and skills they need for meaningful civic participation.

In response to a 2016 health needs assessment that identified a lack of basic health literacy, JEI developed a multi-faceted program to increase access, knowledge, and rights for vulnerable groups. The goal of ATH is to introduce a health educator program that equips nominated community members with knowledge about selected health topics which will then be disseminated to local populations. In accordance with previous studies that have demonstrated the efficacy of these types of programs, we hypothesize that the introduction of a community-driven CHE program will enhance health literacy across local populations and result in measurable improvements in health outcomes.

The objective of this project is to identify health knowledge, attitudes, and behaviors around the populations that would be the focus of a new CHE curriculum. Through this partnership we designed and conducted a survey in low-income settlements around Lagos, Nigeria to assess the baseline level of health literacy and accessibility to resources in these target communities. Determining the level of knowledge prior to the introduction of a CHE program, which allows the quantification of the initiative’s impact after teachings are conducted, and highlighting targeted areas for programmatic quality improvement are the two main purposes for establishing a baseline. Analyzing existing data on community health knowledge gathered by the community will identify the most pressing areas of need that can be addressed through curricular modification. We sought to understand the health literacy status of the overall population as well as differences between targeted demographic groups as to emphasize those interventions that may be most effective in addressing notable health concerns.

## Methods

### Study design

A sectional survey was designed that assessed the health literacy and health status of targeted slum neighborhoods of existing Federation community savings groups. The survey targets key health topics and behaviors that have been shown to reduce the prevalence of diseases common in sub-Saharan African. Topics were selected by community partners as being of perceived importance. Key topics included sanitary behavioral practices, sexual health education, infectious diseases, and access to health services. Collected data underwent descriptive statistics to assess overall performance as well as hypothesis testing through Pearson’s Chi-Squared test to identify differences in knowledge between selected groups. The results will be used to develop a targeted teaching curriculum for CHEs.

### Study setting and survey participants

**Figure 1:**
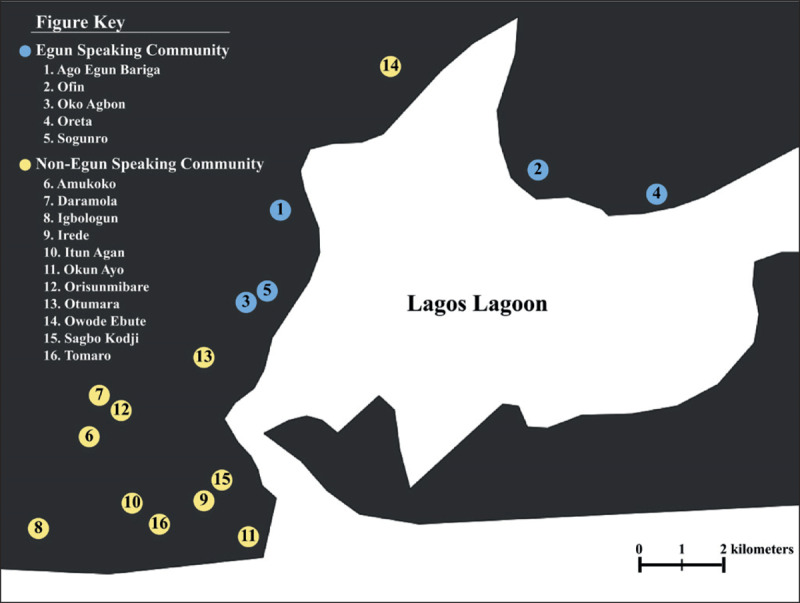
Map of Surveyed Communities around Lagos.

Our survey was conducted in 16 informal settlement communities located around Lagos, Nigeria (Figure 1). Sites selected were areas in which future CHE trainings will take place and are listed in Table 1. Five of these settlements (Ago Egun Bariga, Ofin, Oko Agbon, Oreta, and Sogunro) were predominantly Egun-speaking communities, with the remaining settlements using a language that was predominantly non-Egun. Survey participants were selected based on convenience sampling with snowball effect. Individuals attending community meetings or those who were living in close proximity to planned community events were targeted. Individuals who were able to provide verbal consent and were inhabitants of the community of interest were asked to provide responses to an administered questionnaire.

**Table 1:** Demographics of Survey Population (N = 348).

		Number of Respondents (%)

Age^*^	0–18	9 (2.6)
	19–29	114 (32.8)
	30–39	114 (32.8)
	40–49	66 (19.0)
	50–59	29 (8.3)
	60–69	13 (3.7)
	>70	3 (0.9)
Sex	Male	168 (48.3)
	Female	180 (51.7)
Marital Status	Married	235 (67.5)
	Single	94 (27.0)
	Divorced	10 (2.9)
	Widowed	9 (2.6)
Education Level	None	101 (29.0)
	Primary	91 (26.1)
	Secondary	128 (36.8)
	NCE/NDβ	18 (5.2)
	HND/University^z^	10 (2.9)
Occupation	Trader	128 (36.8)
	Artisan	88 (25.3)
	Fishing	57 (16.4)
	Student	33 (9.5)
	Teacher	10 (2.9)
	Other	32 (9.2)
Community	Ago Egun Bariga^**^	21 (6.0)
	Amukoko	20 (5.7)
	Daramola	21 (6.0)
	Igbologun	20 (5.7)
	Irede	25 (7.2)
	Itun Agan	20 (5.7)
	Ofin**	25 (7.2)
	Oko Agbon^**^	28 (8.0)
	Okun Ayo	5 (1.4)
	Oreta^**^	37 (10.6)
	Orisunmibare	20 (5.7)
	Otumara	21 (6.0)
	Owode Ebute	19 (5.5)
	Sagbo Kodji	25(7.2)
	Sogunro^**^	31 (8.9)
	Tomaro	10 (2.9)

^*^ Mean Age (SE) = 34.7 (0.63). ^**^ Denotes an Egun-speaking community. ^β^ NCE = National Certificate of Education, ND = National Diploma. ^Z^ HND = Higher National Diploma.

### Questionnaire and survey distribution

The community assessment survey contained 37 questions that focused on demographic information, general health topics, and environmental conditions. Question types included yes/no, true/false, and open-ended response. Demographic information collected from surveyed individuals included age, sex, marital status, education level, and occupation. No specific personally identifiable information was used for analysis.

Our questionnaire was developed based on health surveys that had been distributed in settings similar to that of this present study [20,21]. The content of the questionnaire and the distribution process were designed by the Federation in tandem with JEI. The decision to use yes/no questions in assessing health literacy was done in order to obtain targeted answers. For true/false questions, the proportion of respondents who answered correctly was the metric used for data analysis. Those that provided the wrong answer or responded “Don’t Know” were grouped under the “Incorrect” category. Utilizing a dichotomous response structure minimizes relativity in post-survey data interpretation, leading to improved consistency across respondents. In addition, our assessment sought to understand the infrastructural limitations that may prevent knowledge from being translated into behavior. Waste disposal and access to care were key categories used to better understand how the built environment contributes to poor health outcomes.

Data gathering took place from May to July of 2017. Survey results were collected by 28 CHEs who underwent training with a JEI staff member prior to entering communities. Questions provided to interviewers were given in English. Interviewers then read the questions aloud in the native language of those community members they were surveying. Translation from English to the native language was left to the interpretation of the interviewer fluent in the local language in the surveyed areas. Results were recorded on paper and were later translated back into English for analysis. Interviewers initially sought to gather 20 responses per community.

### Statistical methods

Data was compiled in Microsoft Excel (Microsoft Corporation, Redmond WA) and was analyzed using IBM SPSS Statistics for Windows, version 25.0 (IBM Corp., Armonk, N.Y., USA). Descriptive statistics were used to describe frequency of responses. Groups of interest were subdivided according to sex (male v. female), age (youth v. elder), language (Egun v. non-Egun), and education (no education, primary/secondary, and higher education). “Youth” was defined as individuals 30 and under and “Elder” was anyone over the age of 30. This cutoff was determined retrospectively based on the distribution of respondent ages. Individuals were considered to fall under the “higher education” category if they had obtained a National Certificate of Education (NCE), National Diploma (ND), Higher National Diploma (HND), or had attended University. To test associations of variables, Pearson Chi-Square test of independence was used for categorical variables. Chi-Squared tests used a p-level cutoff of 0.05 to determine significance.

### IRB

The project and analysis were reviewed and approved for human subjects exemption by Northwestern IRB.

## Findings

Survey distributors obtained 348 verbal responses from the 16 settlement communities that were sampled. Table 2 summarizes the overall performance of respondents to questions assessing health knowledge. Table 3 lists the remaining survey questions that were centered around health behaviors and health access. Differences in health knowledge between subgroups based on significance testing are listed in Tables 4 and 5. Results are grouped below by health topic and notable findings are highlighted.

**Table 2:** Survey Questions for Health Knowledge Assessment and Percentage Correct.

	True or False Question	Correct^*^	% Correct

9	Rainwater is good for drinking.	False	39.9
10	Water from flowing stream, river or lagoon is safe to drink.	False	73.0
11	Filtering water alone makes it clean for drinking.	False	23.6
12	It is important we wash our hands after stooling.	True	97.1
13	Washing our hands regularly can prevent disease.	True	96.3
14	Family planning helps our family to live healthy.	True	76.7
15	HIV can spread through mosquito bite.	False	50.0
16	You can get HIV when you share spoon, plate or cup with an infected person.	False	52.3
17	A woman who has sex with different men may find it difficult to get pregnant.	False	27.9
18	Vaccine can protect people from disease.	True	81.0
19	Vaccines kill children all the time.	False	77.6
20	The signs of malaria and typhoid can seem alike.	True	55.5

^*^ Incorrect Responses included “Don’t Know”.

**Table 3:** Survey Questions for Health Behaviors and Health Access.

	Question	Responses	N (%)

21*	What are the common sicknesses affecting children in this community?	Malaria	203 (55.3)
		Measles	54 (14.7)
		Cough/Catarrh	42 (11.4)
		Diarrhea	37 (10.2)
		Typhoid	24 (6.5)
		Other	7 (1.9)
22*	Which is the most common sickness affecting adults in this community?	Malaria	138 (36.6)
		Typhoid	70 (18.6)
		Diarrhea	59 (15.6)
		Hypertension	33 (8.7)
		Measles	28 (7.4)
		Cough/Catarrh	12 (3.2)
		Stroke	12 (3.2)
		Diabetes	10 (2.7)
		Other	15 (4.0)
23	How many times have you been sick in the last six (6) months?	0	117 (33.6)
		1	117 (33.6)
		2	72 (20.7)
		3	27 (7.8)
		4	13 (3.7)
		> 5	2 (0.6)
24	What type of sickness did you have last?	Malaria	141 (61.0)
		Typhoid	43 (18.6)
		Cough and Catarrh	14 (6.1)
		Diarrhea	6 (2.6)
		Measles	4 (1.7)
		Hypertension	3 (1.3)
		Stroke	2 (0.9)
		Diabetes	1 (0.4)
		Other	17 (7.4)
25	Where do most people go when they are sick?	Chemist	112 (32.2)
		Health Center	91 (26.1)
		Agbo Seller	52 (14.9)
		Other	93 (26.7)
26	Where do you go when you are sick?	Chemist	116 (33.3)
		Health Center	91 (26.1)
		Agbo Seller	44 (12.6)
		Other	97 (27.9)
27a	Is there a health center nearby?	No	161 (46.3)
		Yes	154 (44.3)
		Don’t Know	33 (9.5)
27b	If yes, how easy is it for you to reach there?	Easy	100 (64.9)
		Difficult	54 (35.1)
28a	Can you get free health services at the health center?	No	80 (51.9)
		Yes	71 (46.1)
		Don’t Know	3 (1.9)
28b	If yes, what services are free?	Malaria	54 (65.1)
		Measles	7 (8.4)
		Typhoid	7 (8.4)
		Diarrhea	5 (6.0)
		Family Planning	2 (2.4)
		Cough/Catarrh	2 (2.4)
		Other	6 (7.2)
29	How do you dispose of your refuse?	Burning	194 (55.7)
		Gutter or Drain	53 (15.2)
		Roadside	23 (6.6)
		Other (Lagoon, Bury)	78 (22.4)
30	Do you participate in regular community environmental sanitation?	Yes	233 (67.0)
		No	115 (33.0)
	If yes, what do you do?	Sweep	109 (46.8)
		General Cleaning	49 (21.0)
		Rake the Gutter	16 (6.9)
		Cutting the Grass	16 (6.9)
		Packing the Gutter	10 (4.3)
		Monitoring	7 (3.0)
		Washing	6 (2.6)
		Other	20 (8.6)
	If no, would you be willing to participate?	Yes	89 (77)
		No	26 (23)
31	Do you have a toilet in your house/compound or otherwise very nearby?	Yes	214 (61.5)
		No	134 (38.5)
32	If yes, what kind of toilet do you use?	Hanging Toilet	108 (50.5)
		Flush Toilet	66 (30.8)
		Pit Latrine	28(13.1)
		Bucket Latrine	6 (2.8)
		Dunghill	5 (2.3)
		Nylon Bag	1 (0.5)
33a	If no, which toilet do you use?	Public Toilet	56 (41.8)
		Nylon Bag	44 (32.8)
		Bush	25 (18.7)
		Dunghill	6 (4.4)
		Other	3 (2.2)
33b	If public/community toilet, which type is it?	Hanging Toilet	41 (73.2)
		Flush Toilet	11 (19.6)
		Pit Latrine	3 (5.4)
		Bucket Latrine	1 (1.8)
34a	Would you pay to use a flush/modern toilet?	Yes	108 (80.6)
		No	26 (19.4)
34b	If yes, how much? (naira)	5	1 (0.9)
		10	21 (19.4)
		20	69 (63.9)
		30	3 (2.8)
		50	14 (13.0)
35	Are there any health problems that result from hanging toilets, bucket latrine, pit latrine, or use of nylon?	No	266 (76.4)
		Yes	78 (22.4)
		Don’t Know	4 (1.1)
36a	Where do you get the water you use?	Borehole	199 (57.2)
		Well	84 (24.1)
		Buy from Mallam	51 (14.7)
		River	4 (1.1)
		Other	10 (2.9)
36b	Is it safe to drink?	Yes	198 (56.9)
		No	150 (43.1)
37	If no, how do you make your water clean for drinking?	Drink “Pure” Water	120 (80.0)
		Boiling	15 (10.0)
		Water Guard	7 (4.7)
		Filtering	4 (2.7)
		Other	4 (2.7)
38	How much would you pay for a jerry can of clean drinkable water?	0	87 (25.0)
		5	20 (5.7)
		10	115 (33.0)
		15	1 (0.3)
		20	61 (17.5)
		25	8 (2.3)
		30	11 (3.2)
		50	37 (10.6)
		60	3 (0.9)
		100	5 (1.4)
39a	Do you wash your hands?	Yes	338 (97.1)
		No	10 (2.9)
39b	If yes, when do you wash your hands?^*^	After Food	159 (47.3)
		Anytime hands are dirty	173 (51.7)
		Before Food	186 (55.6)
		After Toilet	154 (46.6)
		Before Cooking	81 (24.2)
		Other	2 (0.7)
40	What do you use to wash your hand?	Soap and Water	271 (80.2)
		Water Only	67 (19.8)
41	Would you be willing to contribute to help keep a bucket of clean water and soap outside the toilet for children to use?	Yes	304 (87.4)
		No	44 (12.6)
42	Do you get tested for malaria before taking drugs?	No	200 (57.5)
		Yes	148 (42.5)
43	Are your children immunized?	Yes	241 (69.3)
		No	78 (22.4)
		No Children	29 (8.3)
44	Do you practice family planning?	No	245 (70.4)
		Yes	103 (29.6)
45	What changes could your community make to improve the health of the people?	Sanitation	100 (28.7)
		Blank	57 (16.4)
		Clean Water	46 (13.2)
		Better Health Center	36 (10.3)
		Health Advice	28 (8.0)
		Toilet	31 (8.9)
		Other	50 (14.4)

^*^ Respondents were allowed to state multiple responses.

**Table 4:** Survey Questions for Health Knowledge Assessment: Differences among participants according to sex, age, language, and education using Chi-Square Analysis.

		Q9 Water	Q10 Water	Q11 Water	Q12 Hygiene	Q13 Hygiene	Q14 Family	Q15 HIV	Q16 HIV	Q17 Sex	Q18 Vaccine	Q19 Vaccine	Q20 Malaria

Sex	Male (n = 168)	0.39	0.77	0.26	0.96	0.97	0.72	0.51	0.52	0.24	0.82	0.79	0.58
	Female (n = 180)	0.41	0.69	0.21	0.98	0.96	0.81	0.49	0.53	0.31	0.80	0.77	0.53
	p-Value	0.81	0.12	0.26	0.45*	0.47	**0.04**	0.83	0.85	0.16	0.61	0.67	0.41
Age	Youth (n = 153)	0.44	0.80	0.24	0.96	0.96	0.72	0.52	0.59	0.27	0.78	0.75	0.50
	Elder (n = 195)	0.37	0.68	0.24	0.98	0.96	0.81	0.48	0.47	0.28	0.83	0.80	0.60
	p-Value	0.19	**0.01**	0.99	0.30*	0.87	0.06	0.45	**0.02**	0.88	0.27	0.22	0.05
Language	Egun (n = 142)	0.51	0.73	0.18	0.96	0.98	0.77	0.46	0.42	0.25	0.66	0.73	0.58
	Non-Egun (n = 206)	0.33	0.73	0.28	0.98	0.95	0.77	0.53	0.59	0.30	0.91	0.81	0.54
	p-Value	< **0.01**	0.87	**0.03**	0.21	0.19	0.99	0.19	< **0.01**	0.27	< **0.01**	0.06	0.48
Education	None (n = 101)	0.39	0.79	0.15	0.99	0.98	0.79	0.37	0.45	0.17	0.80	0.81	0.50
	1°/2° (n = 219)	0.41	0.69	0.25	0.96	0.96	0.75	0.55	0.51	0.29	0.80	0.74	0.57
	Higher (n = 28)	0.39	0.79	0.43	0.96	0.93	0.79	0.61	0.93	0.57	0.93	0.89	0.64
	p-value	0.94	0.15	< **0.01**	0.40	0.40	0.73	< **0.01**	< **0.01**	< **0.01**	0.25	0.12	0.28

^*^ Indicates Fisher Exact Test was used. *Note*: Cell data (excluding p-values) indicates proportion of correct responses to the specific survey question. A p-value significance threshold of 0.05 was used.

**Table 5:** Chi-Square Test for Independence with Bonferroni Correction (Education).

		Q11	Q15	Q16	Q17

Higher Education v. No Education	p-value	< **0.01**	0.07	< **0.01**	< **0.01**
Higher Education v. 1°/2° Education	p-value	0.12	**0.01**	0.92	0.06
1°/2° Education v. No Education	p-value	0.14	0.99	< **0.01**	**0.01**

### Participant demographics

Table 1 summarizes survey respondent demographics. Among survey participants, 48.3% were male and 51.7% were female. The majority of our participants were married (67.5%), and the average age was 34.7 years. Only 8.1% had any form of higher education, 36.8% reported some form of secondary education, 26.1% had primary education, and 29.0% reported no formal schooling. More individuals were from predominantly non-Egun speaking communities (59.2%) than Egun speaking (40.8%). The most commonly reported occupations were traders (36.8%), artisans (25.3%), fishermen (16.4%), and students (9.5%).

### Drinking water

Our survey found that 39.9% of respondents correctly answered “False” when asked if rainwater is good for drinking, as opposed to 54.6% who incorrectly responded “True” and 5.5% who said that they did not know. Most respondents incorrectly believed that filtering water alone made it clean enough for drinking; only 23.6% of respondents correctly stated that this was false. Most people (73.0%) possessed an understanding that it was unsafe to drink from an open body of water, whether this might be a flowing stream, river, or lagoon.

When knowledge was compared against behavior, we found that community members largely recognized the importance of having a reliable source of clean water for consumption. Most people obtain water from a borehole (57.2%) or a well (24.1%), while others stated that they purchased clean water (14.7%) to use as their primary source for drinking. However, 43.1% of respondents did not consider the water from these protected sources safe to drink. When this subset of individuals was asked how they purify their water, 80.0% said that they drank “Pure Water,” a brand sold in small plastic bags (about 50 mL capacity), and another 10.0% said that they boiled it.

Diarrhea, a disease associated with consuming water from unprotected sources, accounted for 10.2% of childhood illnesses and 15.6% of adult illnesses. Non-Egun speaking communities were less likely than their Egun-speaking counterparts to know that untreated rain water collected from roofs is unsafe to drink (p < 0.01) but were more likely to understand that filtering water alone does not make it clean for consumption (p = 0.03). Young people were more likely to express caution about drinking from open bodies of water than those who were older (p = 0.01), and those with higher education were significantly more likely than those with no education to understand that filtering water alone does not make it suitable for drinking (p < 0.01).

### Hand washing

Community members displayed a strong understanding about the importance of hand washing, as 97.1% of people responded “True” when asked whether it was important to wash hands after stooling. Furthermore, nearly all respondents (96.3%) correctly identified that washing hands regularly can prevent disease.

When asked about their own hand washing practices, respondents overwhelmingly reported that they washed their hands (97.1%). Even more encouraging was that among those who responded “Yes,” 80.2% said that they use both soap and water. The most common times that people washed their hands were after using the toilet (46.6%) and prior to eating (55.6%), and 51.7% said that they washed anytime their hands were dirty. Most people (87.4%) said that they would be willing to assist in ensuring that a constant supply of soap and clean water was left outside of toilets for children to use.

### Waste disposal

When questioned about their waste disposal practices, the most common methods were burning (55.7%) and throwing it in the gutter or drain (15.2%).

When asked about access to a toilet, 61.5% said that they had access to one. Only 30.8% of these individuals used a flush toilet, while 50.5% used a hanging toilet, which involves waste being excreted into an open water source. Among those who did not have access to a personal toilet, 41.8% depended on public toilets, the most common method being hanging toilets, and 32.8% used nylon bags. Most people (76.4%) did not believe that hanging toilets or nylon could lead to health problems despite these resulting in waste being directly introduced into the surrounding environment. However, the decision to use these methods was largely driven by lack of access rather than preference. 80.6% of respondents said that they would be willing to pay to use a modern flush toilet.

The motivation to improve upon existing community sanitation practices was expressed by the majority of people surveyed, with 67.0% responding “Yes” when asked whether they regularly participate in neighborhood clean-up initiatives. Among the 33.0% who did not, 77.4% said that they would be willing to volunteer if given the chance. Sanitation (28.7%) was the most common response to the question that asked what change could be made to improve the health of all people.

### Family planning and reproductive health

While most respondents understood the importance of family planning, there was an incongruence between knowledge and lived behaviors. Most respondents (76.7%) correctly identified that family planning contributes to maintaining a health family but only 29.6% said that they practice it in their own lives. Females were more likely than males to be knowledgeable about this issue (p = 0.04).

Additionally, only 27.9% of surveyed individuals correctly responded “False” to the statement that women may find it difficult to get pregnant if having sex with multiple partners. A significant number of respondents (26.7%) said that they did not know and nearly half (45.4%) incorrectly responded “True”. Chi-Square testing found that individuals with any form of education were significantly more likely to answer correctly than those with no education (p < 0.01 for higher education, p = 0.01 for primary/secondary education).

### Vaccines

81.0% of respondents stated that vaccines can protect people from disease. Knowledge about their protective benefits is widespread, as 77.6% of people answered “false” when asked if vaccines kill children all the time. A large proportion (69.3%) reported that their own children were immunized.

Measles accounted for 14.7% of childhood illnesses in the community. Non-Egun speaking communities were much more likely to understand the protective health benefits of vaccines than Egun communities (91.3% v. 66.2%, p < 0.01).

### Infectious diseases

The survey found numerous lapses in knowledge surrounding HIV. Only half (50.0%) correctly responded “False” when asked if HIV can be spread through a mosquito bite. Chi-Square analysis found that those with higher education were more likely than those with primary/secondary education to respond correctly (60.7% v. 54.8%, p < 0.01).

When respondents were asked whether HIV can be spread through contact with a spoon, plate, or cup, only 52.3% appropriately responded false. Those who were younger, from non-Egun speaking communities, and had more education were more likely to answer correctly.

Malaria was the most common illness to affect children (55.3%) and adults (36.6%), with typhoid being the second most frequently reported ailment in adults (18.6%). When respondents were asked about their most recent illness, malaria (61.0%) and typhoid (18.6%) ranked as the top answers. Due to the high prevalence of these two diseases, the differences in treatment, and the limited resources in these slum communities, it is important that people undergo confirmatory testing to distinguish between the similar clinical presentations of malaria and typhoid. When asked whether the signs of malaria and typhoid can seem alike, 55.5% correctly responded “True” and a large number (19.1%) of people said that they did not know. In practice, less than half (42.5%) of individuals get tested for malaria before receiving treatment.

### Accessibility to care

Most people either did not have a nearby health center (46.3%) or did not know if one was around (9.5%). Among those who had access to a health center, most (51.9%) said that the center did not provide free health services. For those with access to a center that provided free services, malarial medication was the most common complementary treatment offered (65.1%).

Community members utilize a diverse array of sources to access medical care: 32.2% of people go to a local chemist and 14.9% go to an Agbo (traditional medicine) seller. Only 26.1% of people present to a health center when they are sick.

## Discussion

### Key Results

We sought to establish the baseline health literacy in the 16 slum communities that we targeted. Descriptive statistics identified areas of strength and weakness in overall health knowledge while hypothesis testing enabled us to hone in on subtle differences between subgroups. We found that infrastructural deficiencies embedded in the local environment act as potential health hazards and limit access to care.

### Strengths

Our survey identified two key areas where health knowledge had been successfully translated into protective behavioral practices. Nearly all participants understood the importance of handwashing and the large majority recognized the need for vaccinations. This knowledge had been successfully converted into healthy behavioral practices, such as using soap and immunizing children. Given the recent global outbreak of the novel coronavirus disease, the importance of enforcing these practices has taken on heightened importance for vulnerable populations such as the ones covered in our survey [22]. The high reported prevalence of diarrheal illness and measles highlights the continued need to leverage these areas of strength to further improve child health.

Another encouraging sign was the willingness to volunteer in community improvement projects. Nearly all respondents said that they would help to maintain a consistent source of water and soap for children to wash their hands. This inherent sense of responsibility to one’s neighbors should be harnessed by CHEs in future community interventions. Additionally, we found that younger respondents had a higher proportion of correct responses than older community members on questions dealing with water purification and HIV transmission. Targeting the youth as a primary source of prospective CHEs could result in an expedited training process as they already possess a higher baseline of health literacy.

### Areas to target

Despite the high burden of HIV in Nigeria, an alarmingly high number of respondents falsely believed that mosquitoes or contact with shared utensils could transmit the disease. This knowledge deficit was most pronounced in the no education sub-group; thus, this group should be specifically targeted during the curriculum rollout. Other infectious diseases, particularly malaria and typhoid, were also identified as areas of concern. Common clinical symptoms between malaria and typhoid such as fever can make it difficult to distinguish between the two. Restricted access to health centers, unsanitary environments, and a general lack of knowledge result in high prevalence and limited access to treatment. A multifaceted approach that prioritizes education and provides alternative means to obtaining care should be used to address these issues.

Family planning and reproductive health are other topics that should receive greater attention. Many respondents understood the potential benefits of family planning, but a significantly smaller proportion placed these principles into practice. Respondents were also not widely aware of the connection between drinking from unprotected water sources and becoming ill. Failing to properly purify water and poor waste disposal practices may explain the high prevalence of diarrheal illness. Advocating for increased investment in environmental infrastructure, such as upgrading drainage systems and building protected water sources, can supplement changes in behavioral practices to reduce disease burden in the long-term.

Furthermore, we sought to identify interventions that would most effectively reduce disease transmission in a slum setting. Despite a widespread willingness to use modern flush toilets, most people lack access to these and must compensate by using unsanitary open waste disposal methods such as hanging toilets or nylon bags. Educating people about the consequences of open disposal and mobilizing the community to construct public pit latrines can further minimize exposure to pathogens.

Given the high prevalence of malaria in Nigeria that was corroborated by our survey, a future CHE syllabus should prioritize methods of malarial prevention, such as insecticide-treated mosquito nets, and encourage confirmation testing prior to receiving treatment, as the National Malaria Elimination Programme (NMCP) recommends. Emphasizing behavioral changes that reduce the number of spawning sites for mosquitoes, such as proper waste disposal, could be a simple and effective method for improvement. Furthermore, CHEs can be trained to connect their community members to their local health centers so that malaria can be properly diagnosed and treated early.

### Limitations

Our sample is of communities with active Federation groups and may not be representative of all slum communities. Additionally, interviews were conducted by community members rather than trained interviewers which may have introduced bias; however, it is also possible that respondents were more likely to respond truthfully to fellow community members. While questions were derived from prior published instruments and validated internally, there was limited field validation of the yes/no questions used.

### Bias

The advantage of having Federation members spearhead the project is the higher likelihood of obtaining honest responses due to a preformed foundation of trust; however, this approach may have also increased the chance of social desirability bias in some responses. Selection bias may have arisen as a result of the convenience sampling approach used to find participants. We attempted to minimize this by surveying a large number of people from several different communities.

### Future directions

A community health curriculum that addresses the needs of the community will be developed and deployed into the areas where our survey was conducted. Topics that invoked a high proportion of incorrect responses or revealed lapses in understanding will be prioritized. We plan to reinforce learning objectives using quizzes, journals, and games. These alternatives to didactic teaching have been shown to be effective in populations similar to ours and can be used by CHEs to clearly and accurately convey information [23].

## Conclusion

Our survey identified health topics and behavioral practices that should be emphasized in a future training curriculum for CHEs. Assessing baseline health literacy enables us to quantify improvement following the implementation of a community-driven education initiative that aims to reduce the transmission of diseases commonly found in Nigeria. Understanding the physical barriers and the various factors limiting healthy behavioral practices is the first step towards improving health outcomes.

## Data deposition

https://doi.org/10.21985/N2JR2T

## Additional File

The additional file for this article can be found as follows:

10.5334/aogh.2648.s1 Baseline Survey Data. Survey responses to the administered questionnaire.
